# Identification of Candidate Genes Associated With Prognosis in Glioblastoma

**DOI:** 10.3389/fnmol.2022.913328

**Published:** 2022-07-07

**Authors:** Rongjie Li, Qiulan Jiang, Chunhai Tang, Liechun Chen, Deyan Kong, Chun Zou, Yan Lin, Jiefeng Luo, Donghua Zou

**Affiliations:** ^1^Department of Neurology, The Fifth Affiliated Hospital of Guangxi Medical University, Nanning, China; ^2^Department of Radiation Oncology, The Affiliated Hospital of Youjiang Medical University for Nationalities, Baise, China; ^3^Department of Neurosurgery, The Second Affiliated Hospital of Guangxi Medical University, Nanning, China; ^4^Department of Neurology, The Second Affiliated Hospital of Guangxi Medical University, Nanning, China; ^5^Department of Medical Oncology, Guangxi Medical University Cancer Hospital, Nanning, China

**Keywords:** glioblastoma, AEBP1, Cox regression, overall survival, consensus cluster

## Abstract

**Background:**

Glioblastoma (GBM) is the most common malignant primary brain tumor, which associated with extremely poor prognosis.

**Methods:**

Data from datasets GSE16011, GSE7696, GSE50161, GSE90598 and The Cancer Genome Atlas (TCGA) were analyzed to identify differentially expressed genes (DEGs) between patients and controls. DEGs common to all five datasets were analyzed for functional enrichment and for association with overall survival using Cox regression. Candidate genes were further screened using least absolute shrinkage and selection operator (LASSO) and random forest algorithms, and the effects of candidate genes on prognosis were explored using a Gaussian mixed model, a risk model, and concordance cluster analysis. We also characterized the GBM landscape of immune cell infiltration, methylation, and somatic mutations.

**Results:**

We identified 3,139 common DEGs, which were associated mainly with PI3K-Akt signaling, focal adhesion, and Hippo signaling. Cox regression identified 106 common DEGs that were significantly associated with overall survival. LASSO and random forest algorithms identified six candidate genes (AEBP1, ANXA2R, MAP1LC3A, TMEM60, PRRG3 and RPS4X) that predicted overall survival and GBM recurrence. AEBP1 showed the best prognostic performance. We found that GBM tissues were heavily infiltrated by T helper cells and macrophages, which correlated with higher AEBP1 expression. Stratifying patients based on the six candidate genes led to two groups with significantly different overall survival. Somatic mutations in AEBP1 and modified methylation of MAP1LC3A were associated with GBM.

**Conclusion:**

We have identified candidate genes, particularly AEBP1, strongly associated with GBM prognosis, which may help in efforts to understand and treat the disease.

## Introduction

Glioblastoma (GBM) is an aggressive cancer and the most common malignant brain tumor in adults (Wei et al., 2022). GBM involves severe morbidity and it progresses rapidly, leading to extremely poor prognosis (Hassn Mesrati et al., 2020). Treatments include radical surgical resection, chemotherapy and radiation therapy, but they are relatively ineffective, such that GBM patients have the shortest survival among cancer patients (Louis et al., 2016). Indeed, GBM inevitably recurs after surgery and it becomes resistant to therapy, leading to a 5-year survival rate below 5% (Omuro and DeAngelis, 2013; Majc et al., 2021) and a mean survival time of 15 months from diagnosis (Lv et al., 2020).

Rapid and inexpensive gene sequencing has revolutionized our understanding of GBM (Jovcevska, 2020) by identifying, for example, the transcriptional inhibitor adipocyte enhancer binding protein 1 (AEBP1) (Kim et al., 2001) as a potential driver of GBM (Majdalawieh et al., 2020) and various other cancers associated with poor prognosis. Genome-wide association studies have validated 11 single-nucleotide polymorphisms as risk factors for the disease (Wen et al., 2020). At the same time, advances in immunotherapy against other cancers has stimulated research into the GBM immune microenvironment, which turns out to be highly immunosuppressive and a major obstacle to drug-induced killing of tumor cells (Pombo Antunes et al., 2020).

Together, these studies have highlighted the need for comprehensive analysis of genes, gene methylation, and tumor infiltration by immune cells for understanding GBM onset, progression, and treatment (Lukas et al., 2019; Hu et al., 2020; Qin et al., 2020). Therefore the present study examined these questions across five datasets from public databases.

## Materials and Methods

### Data Collection

The gene expression profiles of 145 GBM patients and 5 controls was obtained from The Cancer Genome Atlas (TCGA^1^). Gene expression profiles were also acquired from the datasets GSE16011, GSE7696, GSE50161 and GSE90598 in the Gene Expression Omnibus (GEO^2^). The GSE16011 dataset contains profiling of 276 GBM patients and 8 controls with survival information. The GSE7696 dataset includes profiles from 80 GBM patients and 4 non-tumoral brain samples with recurrence information. The GSE50161 dataset was generated from surgical tumor samples from 34 patients and 13 normal brain samples. The GSE90598 dataset included gene expression profiles from 16 GBM patients and 7 healthy brain tissues. The GSE36278 dataset included methylation profiles of brain tissue samples from 136 GBM patients and 6 controls. Oncomine database and TIMER database were used to identify the expression of genes.

### Identification of Differentially Expressed Genes

The DEseq2 package in R (Love et al., 2014) was used to identify DEGs between GBM patients and controls in TCGA, while the corresponding analysis in the datasets GSE16011, GSE7696, GSE50161 and GSE90598 was performed using the limma package in R (Ritchie et al., 2015). DEGs whose expression differences were associated with *P* < 0.05 were considered significant and analyzed further. DEGs that were up- or downregulated across all five datasets were defined as *common DEGs*.

### Functional Analysis of DEGs

The potential functions of common DEGs were explored by examining their enrichment in Gene Ontology (GO) biological processes and Kyoto Encyclopedia of Genes and Genomes (KEGG) pathways using the clusterProfiler package in R (Yu et al., 2012). *P* < 0.05 was set as the threshold of statistical significance.

Functions or pathways enriched in DEGs were assessed for activation or repression using gene set variation analysis (GSVA) with the GSVA package in R (Hanzelmann et al., 2013). Gene set enrichment analysis (GSEA) was also performed using the clusterProfiler package (Subramanian et al., 2005), and results were displayed using the fgsea package in R.

### Association of DEGs With Overall Survival

DEGs identified in TCGA and the GSE16011 dataset were assessed for their association with overall survival of GBM patients using Kaplan-Meier curves. In addition, Cox regression was used to identify which genes previously linked to survival of GBM patients as well as common DEGs to obtain prognosis-related DEGs. The online tool Metascape^3^ was used to analyze the potential biological functions of prognosis-related DEGs.

### Identification of Candidate Genes for a Prognostic Model

Using the glmnet package in R (Friedman et al., 2010), we performed binomial least absolute shrinkage and selection operator (LASSO) regression to identify common DEGs related to prognosis. This process can compress some coefficients to zero, and then only factors with non-zero coefficients are retained. We sequentially incorporated these factors into the Cox model, stopping inclusion when the area under the receiver operating characteristic curve (AUC) value peaked, at which point the model was considered optimal. AUC was calculated using the pROC package in R (Robin et al., 2011). Survival curves for both groups were plotted using the Kaplan–Meier method.

Survival data were dimensionally reduced using a random forest survival algorithm (Wang et al., 2020), ranked based on factor importance and then filtered for gene signatures. Forest plots were generated by univariate Cox regression analysis using the forestplot package in R. Genes with hazard ratio (HR) > 1 were considered risk factors, HR < 1 were protective factors. The Gene signatures overlapping between the LASSO analysis and random survival forest plots were defined as candidate genes.

### Construction of the Risk Score-Based Prognostic Model

Candidate genes were classified using a Gaussian mixed model (GMM) based on their ability to predict recurrence in the GSE7696 dataset, following a previously described procedure (Hong et al., 2020). Briefly, genes were clustered into mixture models of genes with similar expression patterns. The optimal cluster was selected based on the AUC calculated for each model. Then candidate genes were selected to construct a risk score prognosis model, and GBM patients were divided into low- and high-risk groups based on the median risk score. A prognostic nomogram, constructed using Cox regression, was generated to predict overall survival at three and 5 years based on TCGA dataset. The predictive ability of the risk score prognostic model was assessed in terms of time-dependent AUC, calculated with the timeROC package in R (Blanche et al., 2013).

### DNA Methylation and Somatic Mutations

The cAMP package in R was used to identify differences in methylation between GBM patients and controls in the GSE36278 dataset. Somatic mutations in GBM patients in the TCGA dataset were analyzed using the maftools package in R.

### Immune Cell Infiltration

CIBERSORT^4^ and single—sample GSEA (ssGSEA) in the GSVA package in R were used to assess the levels of immune cell infiltration, and differences in infiltration between GBM patients and controls were calculated using the limma package in R. CIBERSORT were also used in immune cell infiltration for glioblastoma (Kesarwani et al., 2019). We also evaluated potential correlations between candidate genes and immune cell types using Pearson correlation analysis, with significance defined as *P* < 0.05.

### Consensus Clustering, Clinical Characteristics and Drug Sensitivity of GBM Subtypes

The ConsensusClusterPlus package in R (Wilkerson and Hayes, 2010) was used to cluster GBM patients from the GSE16011 database, based on expression of candidate genes. Kaplan–Meier survival curves were compared between the resulting clusters. Potential correlations between patients’ cluster assignments and their clinical information were explored using Pearson correlation.

Drug sensitivity of genes in the clusters of GBM patients, in terms of IC_50_ values, was predicted using the Genomics of Drug Sensitivity in Cancer (GDSC) database^5^ using the pRophetic package in R. Significance was defined as *P* < 0.05. TIDE algorithm (Jiang et al., 2018) was used to predict the responsive to immunotherapy of GBM subtypes.

## Results

### Identification of GBM-Related Genes

**FIGURE 1:**
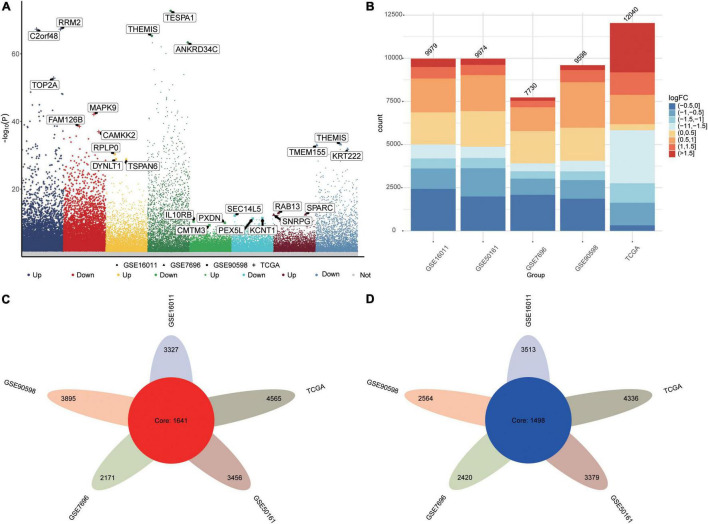
Differentially expressed genes (DEGs) between GBM patients and controls. **(A)** Manhattan plot of DEGs in the datasets TCGA, GSE16011, GSE7696, GSE50161 and GSE90598. **(B)** Statistical bar graph of DEGs in each group. **(C)** DEGs upregulated in GBM across all five datasets. **(D)** DEGs downregulated in GBM across all five datasets.

The flowchart of this study is shown in Supplementary Figure 1. We identified DEGs between GBM and controls in the five datasets as follows: TCGA, 12040 DEGs; GSE16011, 9979 DEGs; GSE7696, 7730 DEGs; GSE50161, 9974 DEGs; and GSE90598, 9598 DEGs (Figures 1A,B). Common DEGs, which were dysregulated in the same direction across all five datasets, amounted to 1,641 upregulated DEGs (Figure 1C) and 1,498 downregulated DEGs (Figure 1D).

### Biological Functions of Common DEGs

**FIGURE 2:**
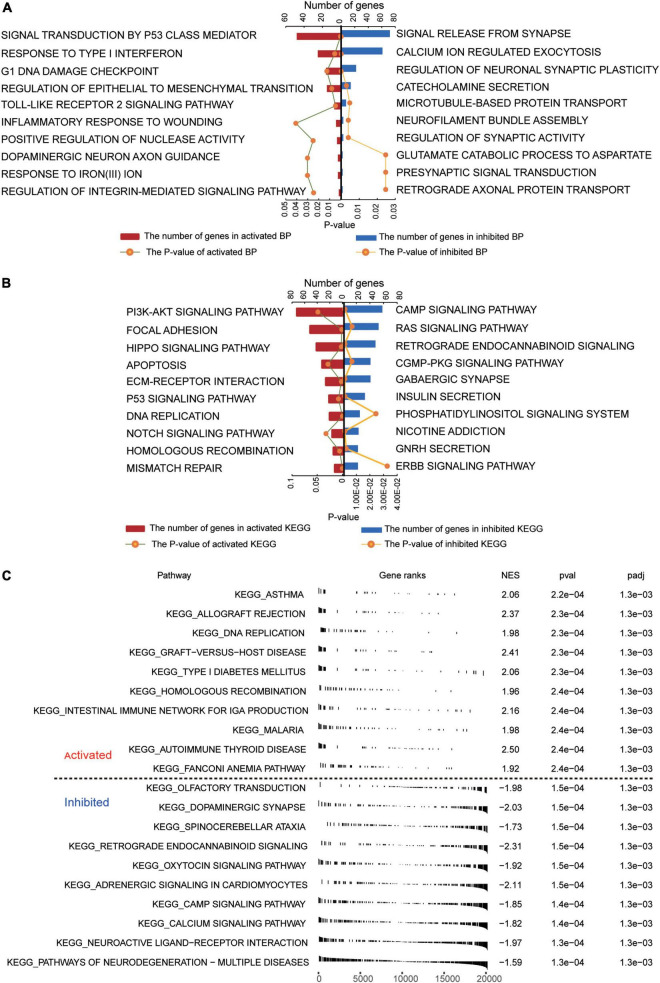
Functional enrichment of common DEGs in GBM. **(A)** Biological processes activated (red) or inhibited (blue) by common DEGs in GBM patients relative to controls. **(B)** KEGG pathways activated (red) or inhibited (blue) by common DEGs in GBM patients relative to controls. **(C)** Activated or inhibited processes in GBM patients relative to controls, based on gene set enrichment analysis.

Several GO biological processes were enriched in common DEGs. The following processes were found to be activated in patients relative to controls: inflammatory response to wounding, response to type I interferon, and signal transduction by p53 class mediator (Figure 2A). Conversely, the following processes were inhibited in patients: calcium ion-regulated exocytosis, regulation of synaptic activity, and signal release from the synapse.

Several KEGG pathways were enriched in common DEGs. The following were activated in patients relative to controls: PI3K-AKT signaling, focal adhesion, and Hippo signaling. Conversely, the following pathways were inhibited in patients: cAMP signaling, RAS signaling, and retrograde endocannabinoid signaling (Figure 2B). GSEA showed activation of asthma and allograft rejection pathways in patients, while it showed inhibition of pathways involving neurodegeneration-multiple diseases and neuroactive ligand-receptor interactions (Figure 2C).

### Prognosis-Related Candidate Genes in GBM

**FIGURE 3:**
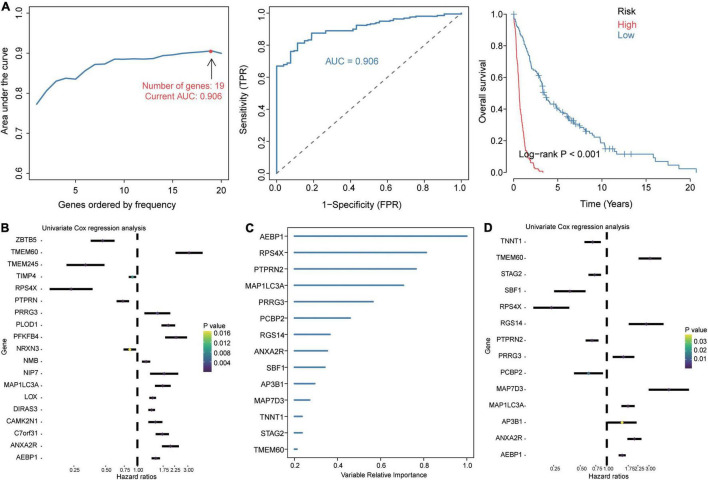
Identification of prognosis-related candidate genes in GBM. **(A)** The 19 gene signatures with the largest AUC values for diagnosing GBM. Signatures were identified using the least absolute shrinkage and selection operator (LASSO) algorithm, receiver operating characteristic curves, and Kaplan–Meier analysis of low- and high-risk groups. AUC, area under the receiver operating characteristic curve; FPR, false positive rate; TPR, true positive rate. **(B)** Forest plots of Cox regression analysis of gene signatures in the LASSO model. **(C)** Importance ranking of 15 gene signatures based on random forest survival modeling. **(D)** Forest plots of Cox regression analysis of gene signatures in the random forest survival model.

Given the extremely poor 5-year overall survival of GBM patients in the GSE16011 dataset (Supplementary Figure 2A), we set out to identify genes associated with survival. Using Kaplan–Meier analysis and Cox regression analysis, we identified 106 common DEGs significantly associated with prognosis. These DEGs were enriched in wound healing, neutrophil degranulation, and extracellular matrix organization (Supplementary Figure 2B). The LASSO model based on 106 genes led to 19 gene signature that predicted GBM with the highest AUC of 0.906 (Figure 3A). A risk score was calculated for each patient using the 19 gene signatures, and the median score was used to stratify patients into low- and high-risk groups, which differed significantly in overall survival. We used univariate Cox regression and forest plots to visualize the distribution of gene signatures (Figure 3B), and we constructed a random forest survival model to select features with the 106 genes. The genes were ranked by relative importance based on the relationship between error rate and number of taxonomic trees, and the most important 15 genes were defined as the optimal signature (Figure 3C). Again, univariate Cox regression and forest plots were used to visualize the distribution of the gene signature (Figure 3D). Six genes overlapped between the 19-gene signature and the final 15-gene signature, and were therefore defined as candidate genes. Forest plots showed that AEBP1, ANXA2R, MAP1LC3A, TMEM60, and PRRG3 were risk factors for GBM, while RPS4X was a protective factor.

### Evaluation of Candidate Genes

**FIGURE 4:**
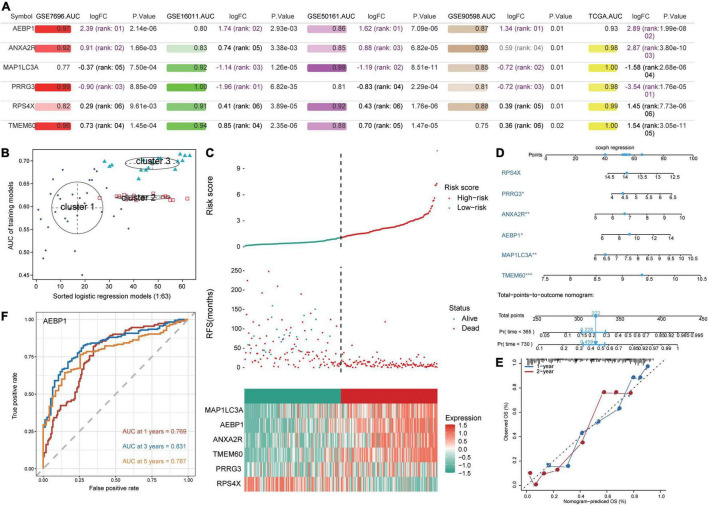
Prediction of GBM patient survival based on candidate genes. **(A)** Areas under the receiver operating characteristic curve (AUCs), fold change in differential expression, and *P* value associated with candidate genes based on the datasets GSE16011, GSE7696, GSE50161, GSE90598, and TCGA. **(B)** Correlation of AUCs with the logistic regression model was identified using a Gaussian mixed model. **(C)** Distribution of candidate gene-based risk scores, recurrence-free survival (RFS) and gene expression levels in the GSE16011 dataset. **(D)** A predictive nomogram was constructed based on candidate genes and the GSE16011 dataset. **(E)** Agreement between nomogram-predicted and observed overall survival (OS) at one and 2 years. **(F)** Receiver operating characteristic curve for AEBP1 to predict OS at one, three, and 5 years.

All six candidate genes had AUCs greater than 0.75 in all five datasets (Figure 4A), and GMM grouped the six candidate genes into three clusters based on their ability to predict GBM recurrence (Figure 4B). Cluster 2 (AEBP1, ANXA2R, MAP1LC3A, PRRG3, and RPS4X) showed the highest AUC for predicting recurrence. Patients were stratified into low- and high-risk groups based on median risk score for the candidate genes, and patients in the low-risk group showed higher recurrence-free survival (Figure 4C). Expression of AEBP1, ANXA2R, MAP1LC3A, TMEM60, and PRRG3 was high in the high-risk group, whereas RPS4X expression was low.

To develop a potentially more clinically useful tool for predicting overall survival in GBM patients, we built a nomogram using multivariate Cox regression (Figure 4D). Calibration plots showed that the nomogram performed well compared with an ideal model (Figure 4E), and the nomogram confirmed the above results about which candidate genes served as risk factors or protective factors.

Using the “timeROC” algorithm, we used the candidate genes to predict overall survival of GBM patients at one, three and five years. AEBP1 led to the highest AUCs (> 0.76) in all three cases (Figure 4F), while each of the other five candidate genes gave AUCs greater than 0.59 in all three cases (Supplementary Figure 3).

### Immune Infiltration in GBM

CIBERSORT showed that M2 macrophages and monocytes were relatively abundant among immune cells in GBM patients in the GSE16011 dataset (Supplementary Figure 4A), while ssGSEA showed infiltration by Th2 cells, T helper cells, neutrophils, and macrophages to be significantly higher in patients than in controls in all five datasets (Supplementary Figure 4B). Conversely, infiltration by NK CD56bright cells was significantly lower in patients than in controls in the five datasets. Infiltrated immune cells fell into three clusters (Supplementary Figure 4C). The candidate genes, especially AEBP1, correlated significantly with T helper cells and macrophages (Supplementary Figure 4D).

### Validation of AEBP1 as a Candidate Marker for GBM

**FIGURE 5:**
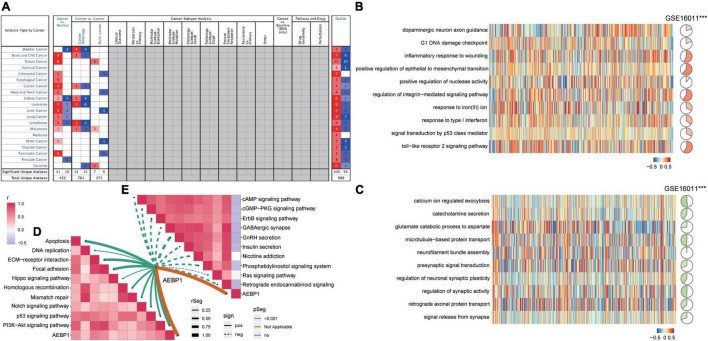
AEBP1 may be a candidate marker for GBM. **(A)** AEBP1 expression in the Oncomine database. Red is high expression and blue is low expression. Numbers represent the number of analyses when the fold change of expression was 2. **(B)** Correlation between AEBP1 and biological processes activated in GBM. **(C)** Correlation between AEBP1 and biological processes inhibited in GBM. **(D)** Correlation between AEBP1 and KEGG pathways activated in GBM. **(E)** Correlation between AEBP1 and KEGG pathways inhibited in GBM.

Analysis of the Oncomine database showed AEBP1 to be upregulated in several types of tumors (Figure 5A), and similar results were found in the TIMER database (Supplementary Figure 5). In addition, according to the expression of AEBP1 and GSVA of functions or pathways, we performed correlation analysis. AEBP1 expression correlated positively with activated biological processes (Figure 5B) and negatively with inhibited processes (Figure 5C). Similarly, AEBP1 expression correlated positively with activated KEGG signaling pathways (Figure 5D) and negatively with inhibited KEGG signaling pathways (Figure 5E).

### Construction of GBM Subtypes

**FIGURE 6:**
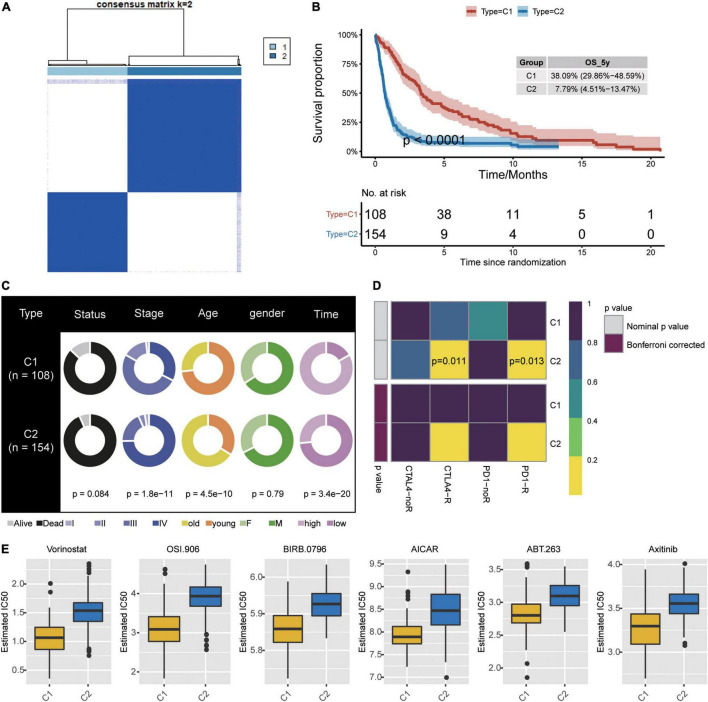
Identification of new subtypes of GBM. **(A)** Consistency clustering based on six candidate genes and GBM patients, generating clusters C1 and C2. **(B)** Kaplan–Meier overall survival (OS) curves for the two clusters. **(C)** Differences in clinical characteristics between the two clusters. **(D)** Differences in predicted response to immunotherapy between the two clusters. **(E)** Predicted drug IC_50_ values that differed the most between the two clusters.

Consistency clustering based on the six candidate genes split GBM samples into class 1 (C1) and class 2 (C2) (Figure 6A). Patients in C1 showed significantly better overall survival (Figure 6B), and the two classes differed significantly in cancer stage, age, and survival time (Figure 6C). The TIDE algorithm predicted that patients in C2 would be significantly more responsive to immunotherapy (*P* < 0.001). Indeed, we predicted C2 to be significantly more likely to respond to anti-PD-1 and anti-CTLA4 treatment when we compared expression profiles of C1 and C2 with those of melanoma patients who responded to immunotherapy (Figure 6D; Roh et al., 2017).

To further explore treatment differences between the two classes of GBM patients, we trained a model with data from GDSC cells to predict IC_50_ for various chemotherapy drugs against patients in C1 or C2. We predicted that 45 drugs would affect the two patient classes differently, particularly the drugs Vorinostat, OSI.906, BIRB.0796, AICAR, ABT.263, and ABT.263 (Figure 6E).

### Mutational and Methylation Landscape of GBM

GBM patients from TCGA showed hypermutated in the genes STAG2, ZBTB5, and NRXN3 (Supplementary Figure 6A), as well as AEBP1 (Supplementary Figure 6B). We screened the GSE36278 dataset for differentially methylated probes (DMPs) between GBM patients and controls (Supplementary Figure 6C), and we identified 61,448 DMPs involving 12,406 genes. When we focused on methylation modifications that affected gene expression in the opposite direction, we identified hypomethylated MAP1LC3A as altered in GBM (Supplementary Figure 6D).

## Discussion

Recent studies have focused on identifying genes that promote or inhibit GBM (Hu et al., 2021; Lin et al., 2021), and we have extended that literature by identifying candidate DEGs associated with prognosis, based on publicly available data. Predicting the response to immunotherapy in patients with different clusters based on candidate genes provides a potential approach for screening immunotherapy patients in the future. The six candidate genes showed a significantly improved prognostic value for GBM patients. In particular, we identified AEBP1 as an oncogene in GBM and as a potential therapeutic and prognostic biomarker.

The DEGs in GBM that we identified appear to be involved in many biological processes and pathways related to nerve function, immune responses, and inflammation. This reflects that tumors act as “wounds that never heal,” hijacking proliferative pathways in wound healing in order to suppress immune responses and induce angiogenesis, all of which favors tumor spread (Dvorak, 1986; Hua and Bergers, 2019). The GO biological processes and KEGG pathways that we found to be activated in GBM concur with previous studies. GBM tumors infiltrate into dense synaptic networks, where they cause axon severing or edema, leading to neuronal degeneration (Lim et al., 2018). GBM progression seems to depend on the PI3K/Akt pathway, which promotes cell proliferation, angiogenesis, migration, invasion and metabolic reprogramming (Su et al., 2021), making it a potential therapeutic target (Lee et al., 2021). Stable focal adhesions, in which integrins connect the cytoskeleton to the extracellular matrix, are essential to maintain the invasive phenotype of GBM cells (Sun et al., 2016; Chen et al., 2018; de Semir et al., 2020). Hippo signaling is strongly associated with poor prognosis in GBM patients (Masliantsev et al., 2021). We found that GBM tissue is extensively infiltrated with neutrophils. Neutrophil infiltration is closely associated with tumor necrosis and predicts poor survival in GBM patients (Yee et al., 2020).

We identified six candidate genes by LASSO and random forest survival modeling that predicted overall survival with AUCs. Gene signatures may also be useful in other cancers, such as the 21-gene panel to evaluate breast cancer recurrence (Oncotype DX) (Wang et al., 2018) and the 18-gene signature to assess recurrence risk in stage II colon cancer patients (ColoPrint) (Kopetz et al., 2015). In addition, we also identified two clusters based on six candidate genes. There are differences between the two clusters in response to immunotherapy. Wang et al. (2021) predicted the clinical response of GBM patients to immunotherapy by TIDE algorithm using gene expression profiles of GBM samples. We also investigated the differences in sensitivity to drugs between patients with different clusters using the GDSC database. Studies exploring drug sensitivity in GBM patients with the GDSC database are also analyzed by Wang et al. (2022).

Five of the six candidate genes that the present work links to GBM have previously been associated with GBM or other cancers. AEBP1 expression has been associated with the poor prognosis of GBM patients (Kalya et al., 2021). Expression of ANXA2R has been shown to correlate inversely with prognosis of glioma patients (Stepniak et al., 2021). High MAP1LC3A expression has been linked to poor prognosis of GBM patients (Wang et al., 2019), perhaps because its upregulation impairs autophagy (Zhang et al., 2016). High expression of PRRG3 is associated with increased risk of prostate cancer (Zhang et al., 2020). Similarly, we are unaware of previous reports linking TMEM60 to GBM or other cancers. Our results identified RPS4X as a protective factor against GBM, which is interesting given that it has been reported to drive the development and metastasis of hepatocellular carcinoma (Zhou et al., 2020). The present study may help focus future research on genes most likely to play key roles in GBM, which may deepen our understanding of the disease and its treatment.

Nevertheless, our findings should be treated with caution in light of important limitations. The first is that our analyses were entirely bioinformatic, so experimental validation is needed. Second, we were unable to correlate gene expression with certain clinicodemographic characteristics of patients, or explore the possibility of clinicodemographic confounding in our analyses. This is because the necessary patient data were often missing from the public databases. In particular, we were unable to stratify patients by severity of GBM, which should be explored in future work.

## Conclusion

We identified six candidate genes that may be prognostic and therapeutic targets for GBM, and we used those genes to build models to predict overall survival and recurrence. These genes and their predictive abilities may help clinicians individualize treatment for GBM patients.

## Data Availability Statement

The original contributions presented in the study are included in the article/Supplementary Material, further inquiries can be directed to the corresponding author/s.

## Ethics Statement

Ethical review and approval was not required for the study on human participants in accordance with the local legislation and institutional requirements. Written informed consent from the patients/participants or patients/participants’ legal guardian/next of kin was not required to participate in this study in accordance with the national legislation and the institutional requirements.

## Author Contributions

RL, QJ, CT, DZ, and JL designed the study and contributed to drafting the manuscript. RL, QJ, CT, LC, CZ, DK, YL, DZ, and JL performed the experiments, and collated and analyzed the data. RL, QJ, CT, JL, and DZ wrote and revised the manuscript. All authors read and approved the final submitted manuscript.

## Conflict of Interest

The authors declare that the research was conducted in the absence of any commercial or financial relationships that could be construed as a potential conflict of interest.

## Publisher’s Note

All claims expressed in this article are solely those of the authors and do not necessarily represent those of their affiliated organizations, or those of the publisher, the editors and the reviewers. Any product that may be evaluated in this article, or claim that may be made by its manufacturer, is not guaranteed or endorsed by the publisher.

## Footnotes

1
https://portal.gdc.cancer.gov/

2
https://www.ncbi.nlm.nih.gov/geo/

3
https://metascape.org/gp/index.html

4
https://cibersort.stanford.edu/

5
www.cancerRxgene.org
