# Supplementary material for: Identification of Candidate Genes Associated With Prognosis in Glioblastoma
Source: Front Mol Neurosci. 2022 Jul 7;15:913328. doi: 10.3389/fnmol.2022.913328 (PMC9302577; doi:10.3389/fnmol.2022.913328)
Supplement: Supplementary file 1 [file Data_Sheet_1.pdf]

Supplementary figures

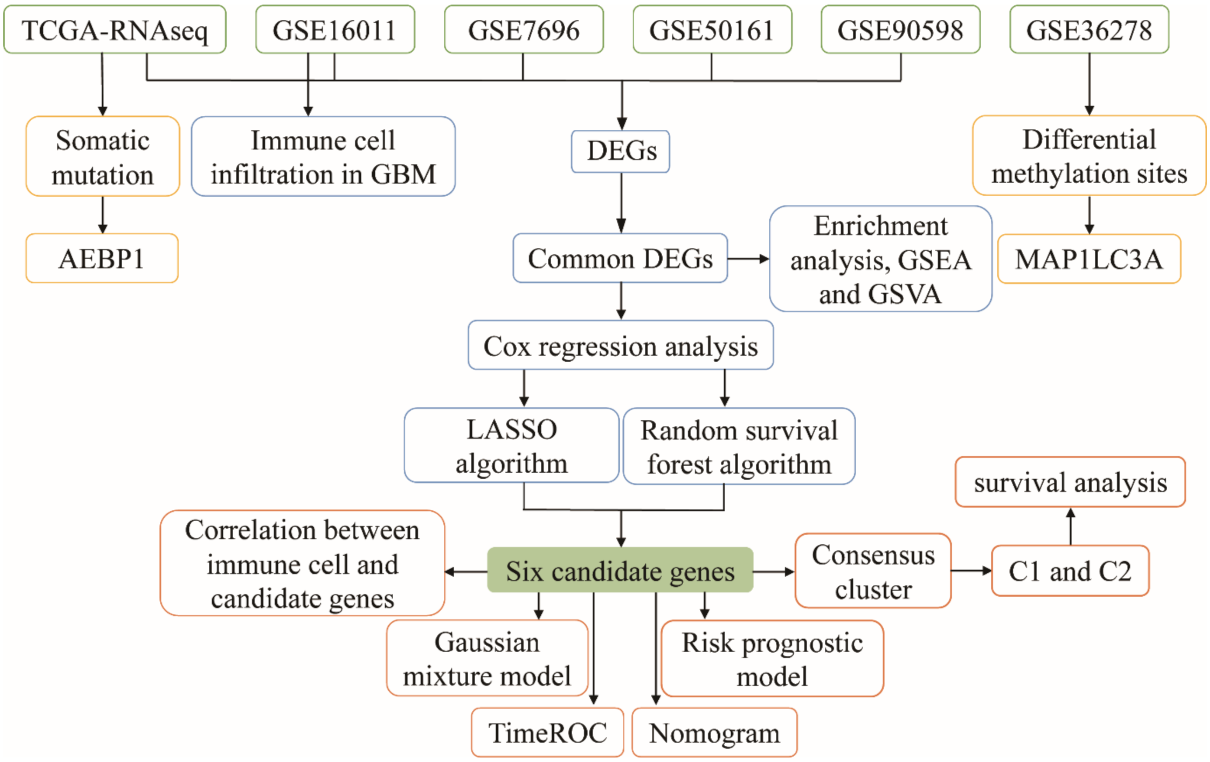

Figure S1. Flow diagram of this study. C1: class 1; C2: class 2; DEG, differentially expressed gene; GBM, glioblastoma; GSEA: gene set enrichment analysis; GSVA: gene set variation analysis; LASSO: least absolute shrinkage and selection operator; ROC: receiver operating characteristic curve; TCGA, The Cancer Genome Atlas.

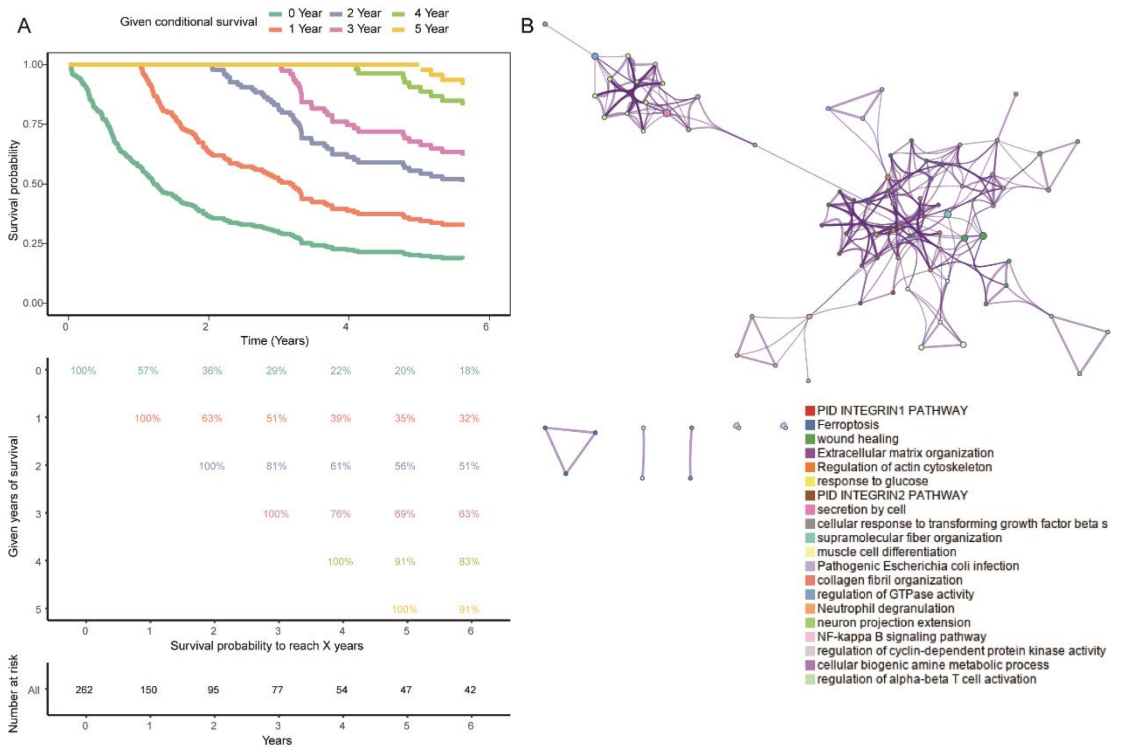

Figure S2. A. Overall survival of GBM patients in the GSE16011 dataset. **B.** Potential biological functions of prognosis‑related genes in GBM, based on Metascape analysis.

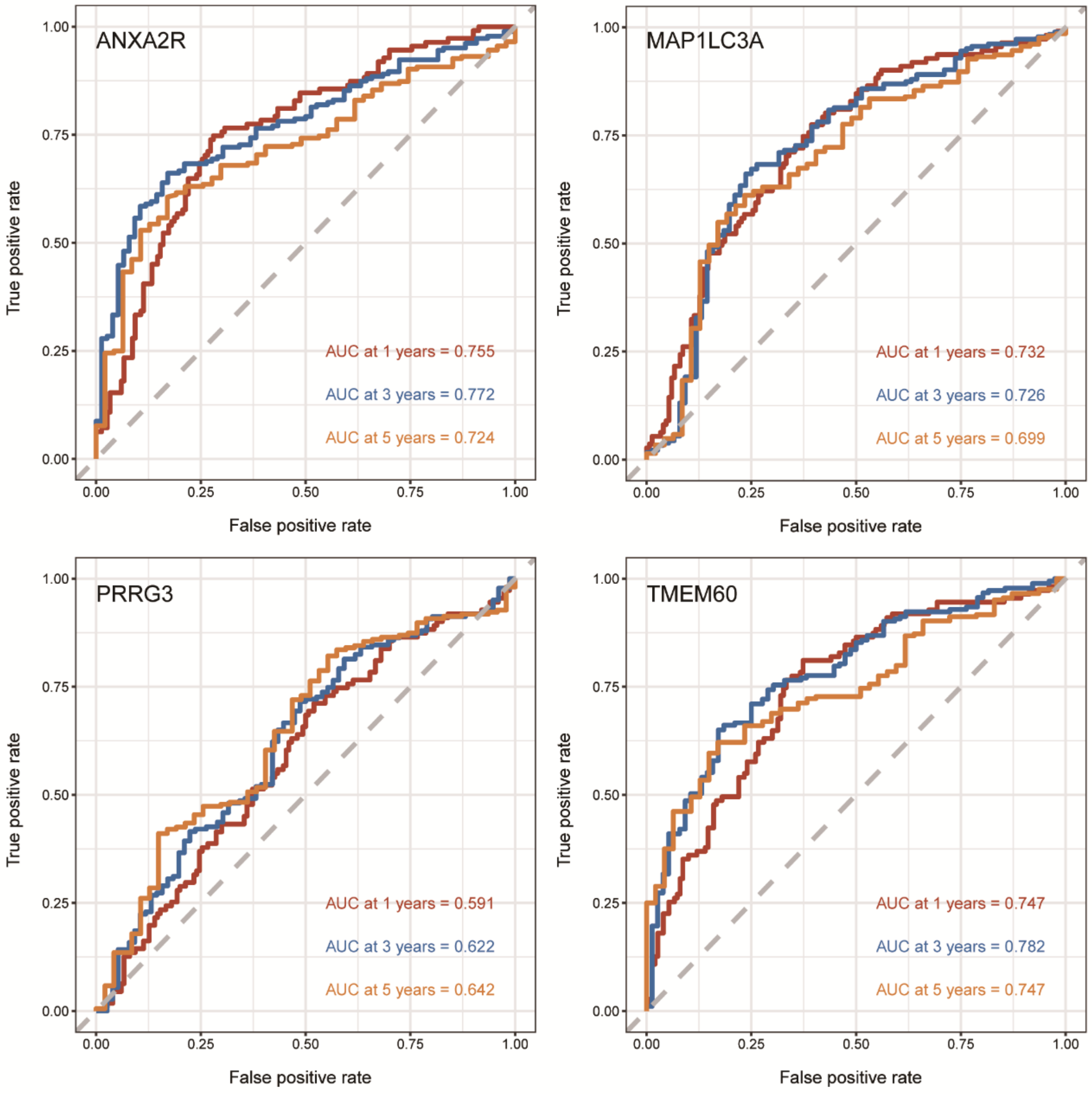

Figure S3. Receiver operating characteristic curves to assess the ability of candidate genes to predict overall survival at one, three, and five years. AUC, area under the receiver operating characteristic curve.

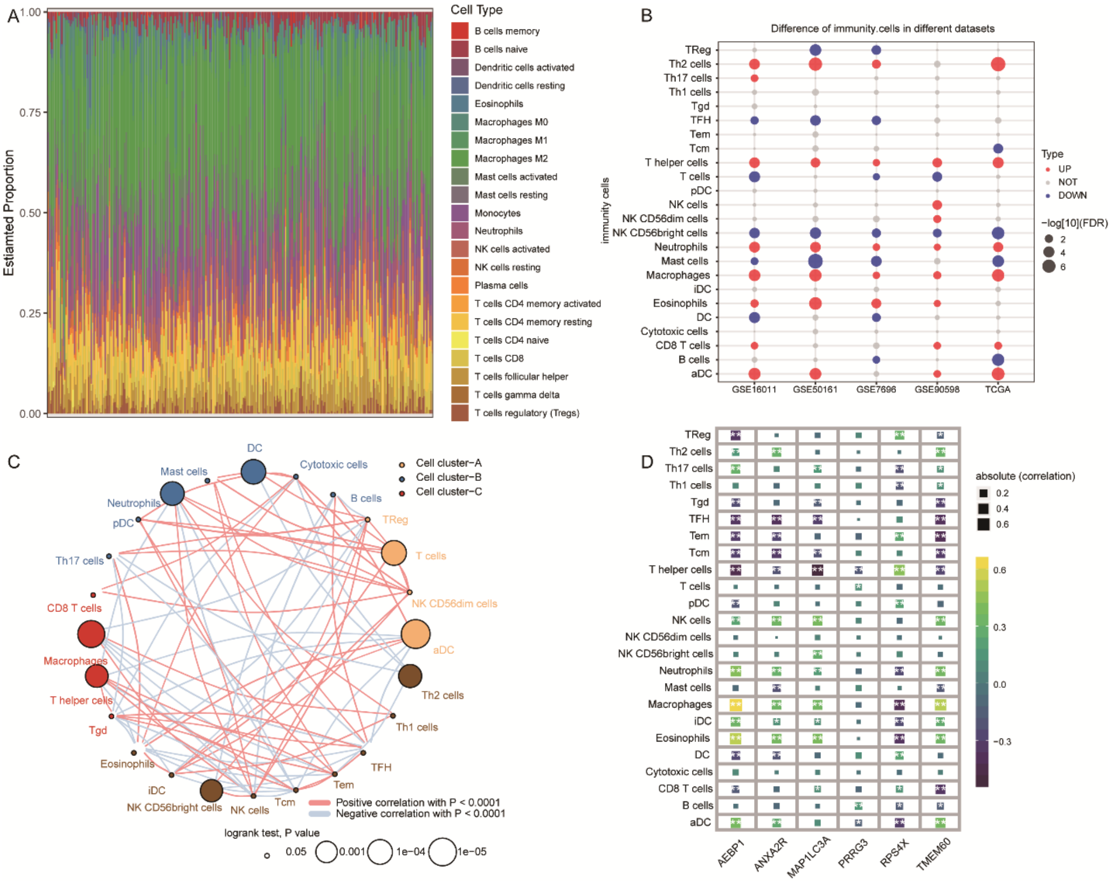

Figure S4. Relationship between immune cell infiltration and candidate genes in GBM. **A.** Proportions of 21 immune cell types, as detected by the CIBERSORT algorithm in GBM patients of the GSE16011 dataset. **B.** Differences in infiltration levels of 24 immune cell types between GBM patients and controls in the datasets GSE16011, GSE7696, GSE50161, GSE90598, and TCGA. **C.** Correlation and clustering results of infiltrating immune cells. **D.** Correlations between candidate genes and infiltrating immune cells. * $P < 0.05$, ** $P < 0.01$.

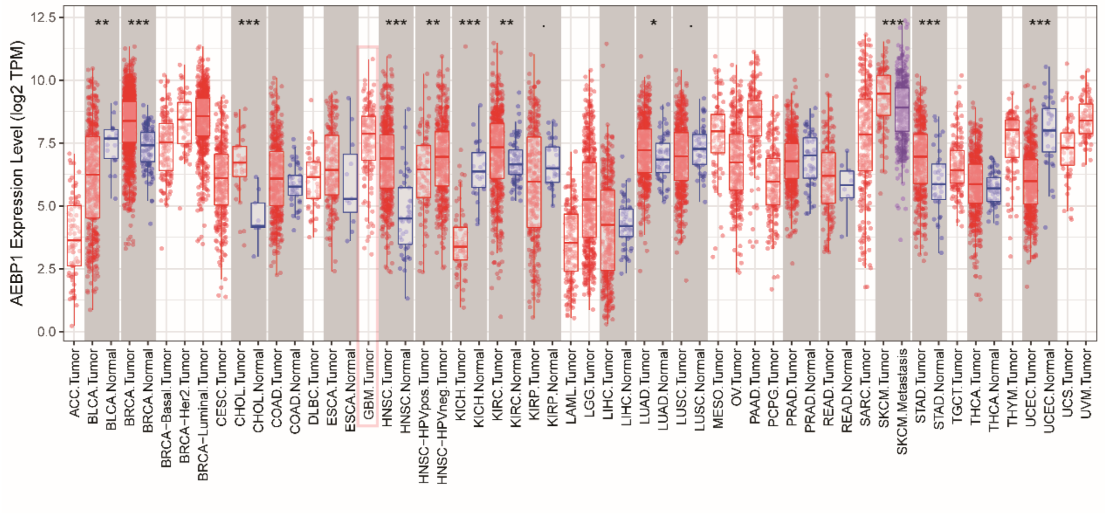

Figure S5. AEBP1 expression in the TIMER database. TPM, transcripts per million. * $P < 0.05$, ** $P < 0.01$, *** $P < 0.001$.

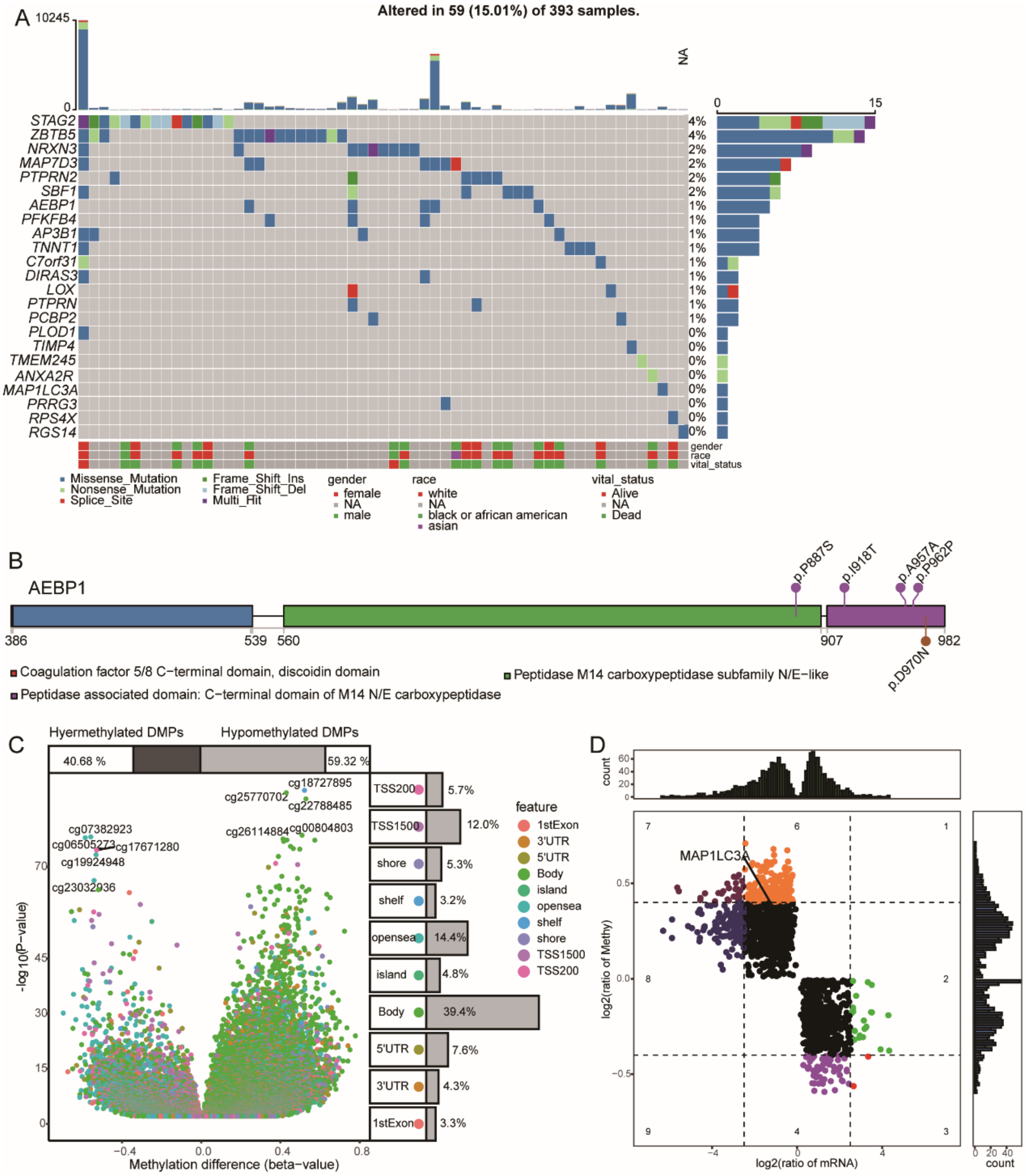

Figure S6. Landscape of mutations and methylation in candidate genes in GBM. **A.** The top 20 somatic mutations of GBM in TCGA. **B.** Mutation spectrum of AEBP1 in GBM patients. **C.** Differentially methylated probes (DMPs) between GBM patients and controls in TCGA dataset. **D.** DBeta and expression of DMPs.
